# Investigating the Effective Factors of Using Personal Protective Equipment from the Perspective of Nurses Caring for COVID-19 Patients: A Cross-Sectional Study

**DOI:** 10.3390/ijerph18157882

**Published:** 2021-07-26

**Authors:** Razieh Mokhtari, Ali Safdari, Davood Hekmatpou, Ali Sahebi, Siamak Moayedi, Mercedes Torres, Mohamad Golitaleb

**Affiliations:** 1 Department of Nursing, School of Nursing, Arak University of Medical Sciences, Arak 3819693345, Iran; razieh.mokhtari91@gmail.com (R.M.); dr_hekmat@arakmu.ac.ir (D.H.); 2 Student Research Committee, Arak University of Medical Sciences, Arak 3819693345, Iran; asafdari.nu@gmail.com; 3 Non-Communicable Diseases Research Center, Ilam University of Medical Sciences, Ilam 6931851147, Iran; ali.sahebi.phd@gmail.com; 4 Department of Emergency Medicine, School of Medicine, University of Maryland, Baltimore, MD 21201, USA; mak.moayedi@som.umaryland.edu (S.M.); mtorres@som.umaryland.edu (M.T.)

**Keywords:** COVID-19, personal protection equipment (PPE), nurse, protective equipment

## Abstract

Considering the importance of appropriate personal protective equipment (PPE) for preventing COVID-19 transmission, the aim of this study was to investigate the factors affecting the use of PPE from the perspective of the nurses caring for COVID-19 patients. This descriptive cross-sectional study surveyed 240 nurses working in the central COVID-19 hospitals of Arak, Iran. Nurses were enrolled in the study by a convenience sampling method. The data collection tool was a validated questionnaire. Data were analyzed by SPSS 16 software using descriptive statistics, analysis of variance (ANOVA), and independent sample *t*-test. Environmental (4.24 ± 0.45), personal (4.16 ± 0.42), and organizational (4.04 ± 0.50) factors all contribute significantly to nursing attitudes about PPE use (*p* < 0.05). The average score, combining all identified factors, was 4.15 ± 0.31. The most influential factor contributing to appropriate use of PPE was environmental, while the least impactful parameters were related to rules and regulations. Environmental factors have the greatest impact on the use of PPE from the perspective of the nurses caring for patients with COVID-19. Managers and healthcare organizations should provide appropriate and adequate PPE to nurses, educate them on proper use, and monitor the process to resolve barriers.

## 1. Introduction

The Association of Infection Control and Epidemiology Specialists supports measures to prevent the transmission of the novel coronavirus from patients to healthcare workers (HCWs) [1,2]. Strict adherence to infection prevention guidelines is a critical component of efforts to stop the spread of infectious and contagious diseases such as SARS-CoV-2 to healthcare personnel [2,3]. A study conducted in China showed that approximately one-third of those infected with COVID-19 were HCWs [4]. In Italy, 10% of healthcare providers contracted the virus, 3% of whom died [1]. This highlights the importance of providing appropriate personal protective equipment (PPE) for HCWs in order to prevent transmission in the healthcare environment [5,6].

Nurses are an essential component of the frontline team caring for COVID-19 patients. Their service is vital to the care of the sick and further efforts to end the pandemic [2,7]. The International Council of Nurses has recognized the key role of nurses in the treatment and care of patients with COVID-19 [8]. Considering the highly infectious nature of the disease and the dire consequences of HCW infections for healthcare infrastructure, it is important to pay close attention to the use of PPE [5,8].

The provision of adequate PPE to nurses has been a significant challenge throughout this pandemic. High cost, limited supply, and high rates of consumption have all contributed to PPE shortages experienced by HCWs worldwide. Shortages in PPE have caused great concern among HCWs regarding their safety and protection [8,9].

In a recent review, factors such as low skill, lack of training, insufficient access to PPE, and environmental factors were noted as barriers to the use of PPE by nurses [10]. In addition, personal characteristics including beliefs, attitudes, and values and organizational factors such as communication, training, performance feedback, and acceptance among colleagues or managers have been shown to influence nurses’ rates of compliance with self-protection behaviors [8,10].

Previous studies have demonstrated that nurses who care for patients with a novel infectious disease (such as severe acute respiratory syndrome (SARS) or H1N1) may be unaware of the most up-to-date information regarding safe patient care and are ill-equipped with PPE [11,12]. The experience of Saudi nurses caring for Middle East respiratory syndrome (MERS) patients also showed that the nurses lacked adequate knowledge about the disease and were more vulnerable to contracting the virus [12]. Prior to the current COVID-19 pandemic, most recent studies regarding best practices for infection control of HCWs were based on lessons learned during the MERS, SARS, and H1N1 outbreaks [13,14,15]. Nursing compliance with preventive behaviors against respiratory infectious diseases such as SARS and H1N1 has been shown to be influenced by their level of knowledge [16,17], attitudes toward the disease [18], and risk perception [19]. To the best of our knowledge, no study has assessed the impact of individual, organizational, and environmental factors on the use of PPE by nurses caring for patients with COVID-19. We aimed to investigate factors affecting the use of PPE with a focus on nursing perspectives.

## 2. Methods

**Figure 1:**
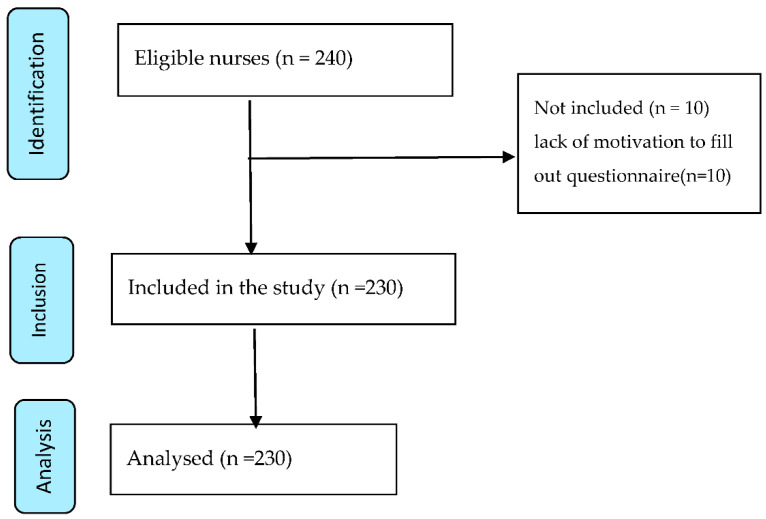
Flow diagram of the selection of study based on STROBE.

This was a descriptive cross-sectional study. The study population consisted of nurses caring for COVID-19 patients in the hospitals affiliated with Arak University of Medical Sciences. All nurses working in the COVID-19 care centers of Valiasr (n = 110) and Ayatollah Khansari (n = 130) hospitals were enrolled via the census sampling method (Figure 1). The study was approved by the Ethics Committee of the university and researchers obtained the permission of hospital directors and head nurses. The research team visited the target wards, spoke to the nurses individually and in groups, and asked eligible nurses to complete this anonymous questionnaire. This occurred during three working shifts (morning, evening, and night). Participants were assured about the confidentiality of their responses and the voluntary nature of the study. Inclusion criteria were having at least a bachelor’s degree in nursing and at least six months of work experience. The exclusion criterion was an unwillingness to participate. The data collection tool, a paper-based questionnaire, was handed to eligible nurses and placed by the researcher into a folder after completion. The time needed to complete the questionnaire was 10 min. The data were collected from 5 October to 15 November 2020. For confidentiality, the questionnaires were filled out anonymously without any identifying data. The content validity of the questionnaire was confirmed by 10 nursing research experts. The content validity ratio (CVR) and the content validity index (CVI) of the checklist were calculated as 0.71 and 0.94, respectively. Moreover, the total reliability of its items was approved by a Cronbach’s alpha of 0.88 (0.79 for environmental factors, 0.96 for organizational factors, and 0.90 for individual factors). The reliability was also confirmed based on calculating the correlation coefficient index (0.81) through a test–retest method in a pilot study of 15 nurses with an interval of 10 days.

The questionnaire consisted of two parts. The first part included demographic information (gender, age, marital status, education, position, work experience, and previous attendance at any PPE training workshop). The second part consisted of 26 statements about the environmental (4 questions), organizational (9 questions), and individual (13 questions) factors affecting the respondent’s use of PPE. The statements were scored based on a 5-point Likert scale (strongly agree = 5, agree = 4, no opinion = 3, disagree = 2, and strongly disagree = 1). Using this 5-point scale, the hypothetical average score of the population was assumed to be 3 (i.e., the middle point = the option of “no opinion”) during data analysis. The mean score of each item and dimension was determined separately and compared with the hypothetical mean. A mean score of higher than 3 was regarded as above the average, while a mean score of less than 3 was considered below the average. To calculate the average score of each category, the scores of all questions were summed up and then divided by the total number of questions in that domain.

The data were analyzed by SPSS software (version 16) using descriptive statistics. The one-way ANOVA test was used to determine any significant differences when comparing the scores of more than two groups, and the independent sample Student *t*-test was used to examine the mean score differences between two-state variables.

## 3. Results

Out of 240 distributed questionnaires, 230 were returned, for a response rate of 95.8%. Table 1 shows that 195 nurses (84.4%) were female and 35 (15.2%) were male. Overall, 219 nurses (95.2%) had a bachelor’s degree, and 11 (4.8%) had a master’s degree. Regarding position, the majority of participants (187, 81.3%) were clinical nurses, while only two (0.9%) were supervisors. Most respondents were married (152, 66.1%). The mean duration of work experience was 12.38 ± 6.22 years, and the mean age was 37.23 ± 7.13 years. More than half of the nurses (126, 54.8%) had participated in PPE training workshops.

The factors affecting the use of PPE by nurses participating in this survey were environmental (4.24 ± 0.45), individual (4.16 ± 0.42), and organizational (4.04 ± 0.50), listed in order of expressed importance. The average score for all categories was 4.15 ± 0.31. Considering homogeneous variances and normal distribution based on the Kolmogorov–Smirnov test (*p* < 0.05), the univariate *t*-test rendered significant results for all of the environmental, organizational, and individual factors (*p* < 0.05). Therefore, as the observed averages were significantly greater than the hypothetical mean (i.e., 3), these factors were found to have a substantial impact on decision making (Table 2).

After examining the homogeneity of variances using Levene’s test (*p* < 0.05), a *t*-test was used to compare the mean scores obtained based on gender, education, marital status, and previous participation in a PPE training workshop (Table 3). Nurses with a bachelor’s degree placed more importance on personal factors contributing to their decisions regarding PPE use when compared with nurses who held a master’s degree, although this difference was not found to be highly significant (*p* = 0.08). The results of the *t*-test show that nursing compliance with the use of PPE was significantly associated with female gender (*p* = 0.05). An increased importance of environmental factors was significantly associated with being married (*p* < 0.05).

The results of the analysis of variance of the total score after confirming the homogeneity of variances based on Levene’s test (*p* < 0.05) show that the highest and lowest total mean scores were related to the charge nurse and nurse supervisor positions, with mean scores of 129.80 and 65.50, respectively (Table 4).

## 4. Discussion

HCWs are at the frontline of fighting COVID-19 and should use PPE to protect themselves against the disease. Conversely, wearing PPE increases their stress and workload [20]. Our results show that environmental factors were the most impactful on nurses’ decisions regarding the use of PPE, while individual preferences carried less weight. A study from Pakistan showed that a lack of availability and inappropriate use of PPE were among the most notable factors contributing to the transmission of COVID-19 disease to HCWs [21]. Furthermore, an Italian study showed that proper education regarding the use of PPE was just as important as providing adequate supplies [22]. Adequate and appropriate access to PPE reduces the incidence of mental health disorders such as depression and anxiety in nurses [23]. Therefore, in addition to access to PPE, HCWs should receive the necessary and appropriate education to use this equipment safely. Our results show a relatively low importance of personal factors, such as knowledge, attitudes, and beliefs, on the use of PPE by nurses. A study in Nepal showed that it is possible to improve attitudes and safety performances by disseminating accurate information about COVID-19 transmission and infection [24,25]. It is likely that increased efforts aimed at educating nurses regarding COVID-19 transmissibility and infection could improve their attitudes toward, and compliance with, recommended PPE use.

Organizational factors also affected nurses’ attitudes toward PPE to some degree. Healthcare organizations should consider continuously training their personnel to use PPE as a part of their COVID-19 pandemic response programs [26]. Furthermore, Delgado et al. showed that supporting HCWs should be among the strategic priorities of healthcare systems during this pandemic [27]. Ahmed et al. also showed that providing HCWs with PPE is essential, and hospital managers and governments should implement measures to guarantee their access [28]. Overall, our study showed that the factors that had the greatest impact on the views of the nurses caring for patients with COVID-19 regarding the use of personal protective equipment were as follows: environmental factors such as availability of PPE, lack of barriers to safe work practices, and cleanliness and order of the workplace; organizational factors such as feedback from supervisors and safety officers regarding the use of PPE, providing constructive and continuous education to nurses on the use of PPE, addressing staff shortages, implementing quarantine and isolation policies, limiting time for patient care, and high work pressure and workload; personal factors such as believing in the effectiveness of PPE, perception of the organization’s safety requirements, the impact of mental norms on the use of PPE, knowledge of coronavirus transmission routes, knowing how to use PPE, understanding the risk of contracting the COVID-19, and setting an example for colleagues by using PPE.(Table 5).

Ultimately, healthcare systems have an important role in maintaining an adequate supply of PPE for nurses during the COVID-19 pandemic. If this role is neglected, organizations face declining quality of care, increased risk to staff, worsening levels of burnout, and an overall compromise in their efficiency and performance.

## 5. Limitations

A limitation of our study was the relatively low sample size. This can potentially reduce the generalizability of the results. One way to address this would be to pursue larger, multicenter studies in the future. In addition, if respondents were concerned regarding the confidentiality of their answers, they may have provided answers that were less critical of their workplace or organization. This may have altered the results to show a greater support for the use of PPE than in actual practice. This may also have resulted in less critique of the provision and organizational support for PPE than truly experienced. Counteracting this effect, however, may also be a concern for stigma against personal beliefs that conflict with hospital policy, therefore making participants less likely to express their personal beliefs if at odds with those of the organization. Finally, the cross-sectional design of the study may limit our results.

## 6. Conclusions

Environmental factors had the greatest impact on the use of PPE from the perspective of the nurses caring for COVID-19 patients. Managers and healthcare organizations should provide appropriate and adequate PPE to nurses, educate them on proper use, and monitor the process to resolve barriers.

## Figures and Tables

**Table 1:** Demographic characteristics of study participants.

Variables		*N* (%)
Gender	Female	195 (84.8%)
	Male	35 (15.2%)
Education	Bachelor’s degree	219 (95.2%)
	Master’s degree	11 (4.8%)
Marital status	Single	78 (33.9%)
	Married	152 (66.1%)
Previous participation in PPE training workshops	Yes	126 (54.8%)
	No	104 (45.2%)
Position	Nurse	187 (81.3%)
	Shift’s chief	32 (13.9%)
	Head nurse	9 (3.9%)
	Supervisor	2 (0.9%)
Age in years (mean ± SD)		37.23 ± 7.13
Work experience in years (mean ± SD)		12.38 ± 6.22

**Table 2:** Univariate *t*-test for the mean score of the factors affecting nursing compliance with the use of PPE (cut off point = 3).

Dimensions	Mean	SD	*p*	T	df	Deviation from Hypothetical Mean
Environmental factors	4.24	0.45	0.001	41.48	229	1.24
Organizational factors	4.04	0.50	0.001	31.38	229	1.04
Personal factors	4.16	0.42	0.001	41.07	229	1.16
All Categories	4.15	0.31	0.001	56.18	229	1.15

**Table 3:** Comparison of the mean scores of the factors affecting nurses’ compliance with using PPE based on gender, marital status, education, and previous participation in a PPE workshop.

Variables		Environmental Factors	Organizational Factors	Personal Factors	Average of All Factors
Gender	Female	4.24 ± 0.44	4.04 ± 0.48	4.17 ± 0.42	4.15 ± 0.30
	Male	4.24 ± 0.53	4.08 ± 0.60	4.06 ± 0.42	4.13 ± 0.35
*p* *		0.13	0.26	0.18	0.05
Education	Bachelor	4.23 ± 0.45	4.04 ± 0.50	4.17 ± 0.42	4.14 ± 0.31
	Master	4.56 ± 0.31	4.18 ± 0.62	3.94 ± 0.48	4.23 ± 0.32
*p* *		0.16	0.37	0.08	0.38
Marital Status	Single	4.15 ± 0.47	4.07 ± 0.48	4.20 ± 0.39	4.14 ± 0.30
	Married	4.29 ± 0.43	4.03 ± 0.51	4.13 ± 0.44	4.15 ± 0.31
*p* *		0.02	0.55	0.22	0.84
Participation in PPE workshop	Yes	4.28 ± 0.44	4.08 ± 0.47	4.19 ± 0.41	4.18 ± 0.29
	No	4.19 ± 0.46	4.00 ± 0.53	4.10 ± 0.32	4.10 ± 0.32
*p* *		0.95	0.60	0.16	0.70

* Independent *t*-test.

**Table 4:** The mean scores of the factors affecting the compliance of nurses with using PPE according to the nurses’ positions.

Position	Mean	SD	Mean of Degree	*p*
Nurse	4.14	0.29	113.77	0.59
Shift’s chief	4.22	0.40	129.80	
Head nurse	4.14	0.21	111.67	
Supervisor	3.98	0.18	65.50	
*p* = 0.13	Df1 = 3		Df2 = 226	F = 1.88

**Table 5:** Frequency, percentage, mean, and standard deviation of nurses’ responses to the factors affecting the use of personal protective equipment.

Number	Factor Impacting the Use of PPE	High and Very High	Moderate	Low and Very Low	Mean	SD
1	The availability of PPE	215 (93.5%)	15 (6.5%)	0	4.53	0.61
2	The lack of barriers to safe work practices	210 (91.3%)	17 (7.4%)	3 (1.3%)	4.31	0.66
3	The cleanliness and order of the workplace	204 (88.8%)	21 (9.1%)	5 (2.1%)	4.33	0.75
4	The patient’s clinical course deterioration	146 (63.7%)	72 (31.1%)	12 (5.2%)	3.79	0.86
5	The support of managers about the implementation of safety procedures	152 (66.1%)	63 (27.4%)	15 (6.5%)	3.76	0.88
6	Feedback from supervisors and safety officers regarding the use of PPE	168 (73%)	48 (20.9%)	14 (6.1%)	3.90	0.90
7	Providing constructive and continuous education to nurses on the use of PPE	190 (82.6%)	36 (15.7%)	4 (1.7%)	4.20	0.76
8	Staff shortage for patient care	188 (81.7%)	36 (15.7%)	6 (2.6%)	4.10	0.77
9	Managers’ expectations on the use of PPE	178 (77.4%)	34 (14.8%)	18 (7.8%)	4.00	1.04
10	The impact of the workplace prevailing safety practices on using PPE	189 (82.2%)	34 (14.8%)	7 (3%)	4.09	0.80
11	Implementing quarantine and isolation policies	194 (84.7%)	30 (12.7%)	6 (2.6%)	4.12	0.76
12	Limited time for patient care	192 (83.4%)	29 (12.7%)	9 (3.9%)	4.13	0.80
13	High work pressure and workload	191 (83.1%)	27 (11.7%)	12 (5.2%)	4.09	0.85
14	Believing in the effectiveness of PPE to prevent infectious disease, such as COVID-19 transmission	208 (90.5%)	16 (6.9%)	6 (2.6%)	4.26	0.72
15	Perception of the organization’s safety requirements	209 (91%)	17 (7.3%)	4 (1.7%)	4.22	0.69
16	The impact of mental norms on the use of PPE	215 (93.5%)	12 (5.2%)	3 (1.3%)	4.32	0.65
17	Having knowledge of coronavirus transmission routes	212 (92.3)	11 (4.7%)	7(3%)	4.29	0.71
18	Knowing how to use PPE	211 (91.8%)	15(6.5%)	4 (1.7%)	4.27	0.66
19	Understanding the risk of contracting the COVID-19	208 (90.5%)	22 (9.5%)	0	4.29	0.63
20	Believing in a reduction in the quality of patient–nurse communication when using PPE	165 (71.7%)	42 (18.3%)	23 (10%)	3.89	0.97
21	Believing in a reduction of agility in patient care when using PPE	183 (79.6%)	38 (16.5%)	9 (3.9%)	4.06	0.79
22	Previous infection of self, or colleague, with the coronavirus or other infectious diseases	207 (11.8%)	20 (86.9%)	3 (1.3%)	4.23	0.65
23	Setting an example for colleagues by using PPE	208 (90.5%)	21 (9.1%)	1 (0.4%)	4.28	0.64
24	Patients’ expectations about nurses using PPE	172 (74.9%)	45 (19.5%)	13 (5.6%)	4.02	0.87
25	A positive attitude toward the protective effect of PPE	206 (89.6%)	24 (10.4%)	0	4.21	0.61
26	Valuing personal judgment over organizational policies	138 (60.1%)	67 (29.1%)	25 (10.8%)	3.68	0.94

## Data Availability

The dataset analyzed during the current study is available from the corresponding author upon reasonable request.
